# 1588. Weight Change among People Living with HIV who switch from Oral Antiviral Therapy to Long Acting Injectable Cabotegravir/Rilpivirine in a University-Based Clinic

**DOI:** 10.1093/ofid/ofad500.1423

**Published:** 2023-11-27

**Authors:** Lukas McNaboe, Julia Kostka, Alvaro Ayala, Dorothy Wakefield, Lisa M Chirch

**Affiliations:** University of Connecticut, New Britain, Connecticut; University of Connecticut, New Britain, Connecticut; University of Connecticut, New Britain, Connecticut; University of Connecticut, New Britain, Connecticut; University of Connecticut School of Medicine, Farmington, CT

## Abstract

**Background:**

Integrase strand transfer inhibitors (INSTIs) are a key component of antiretroviral therapy (ART) for HIV treatment, but associated weight gain limits their use in some patients. This study measures weight in people living with HIV (PLWH) who switched to long-acting injectable (LAI) cabotegravir-rilpivirine (CAB) from oral ART regimens.

**Methods:**

This single-center retrospective cohort study compared weight in patients on oral ART regimens, largely INSTI-based, versus those who switched to LAI CAB over 12 months. Demographics, ART, and clinical parameters were collected at the initiation of CAB (T0), 2 months (T1), 6 months (T2), and 12 months before and after the switch (T3) [table 1]. Three groups were compared: patients who remained on oral INSTI (Group 1), patients before switching to LAI CAB (Group 2), and patients after switching (Group 3). Group 3 on LAI CAB was compared to both Group 2 (the same patients prior to switching), as well as to Group 1. Descriptive statistics were calculated for all variables, means and standard deviations for continuous variables, and frequencies and percentages for categorical variables. SAS 9.4 software was used for statistical analysis.

**Figure:**
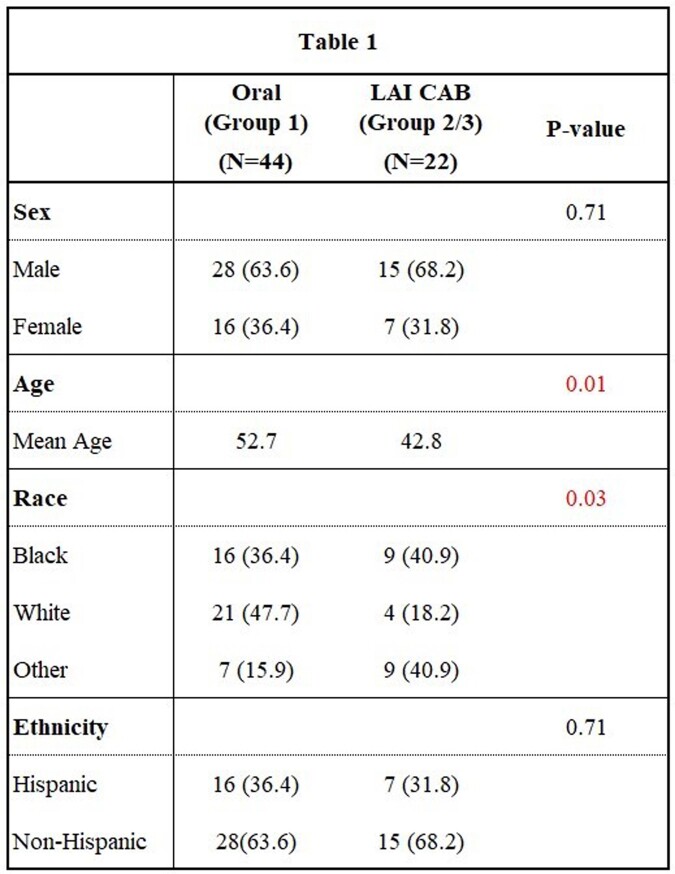

Baseline characteristics of patients on oral-INTI based regimens versus patients on LAI CAB.

**Results:**

A total of 22 patients on CAB and 44 patients on oral INSTI-based regimens met inclusion criteria. Baseline characteristics between cohorts were similar [table 1]. All patients in group 1 and most patients in group 2 were on oral INSTIs (78.2%). A significant difference in weight change was observed between groups 1 and 3 at 12 months [figure 1]. No significant weight difference was observed between groups 2 and 3 at 6 months. Estimated glomerular filtration rate (eGFR) was lower in group 1 [table 2].

Figure 1

**Figure:**
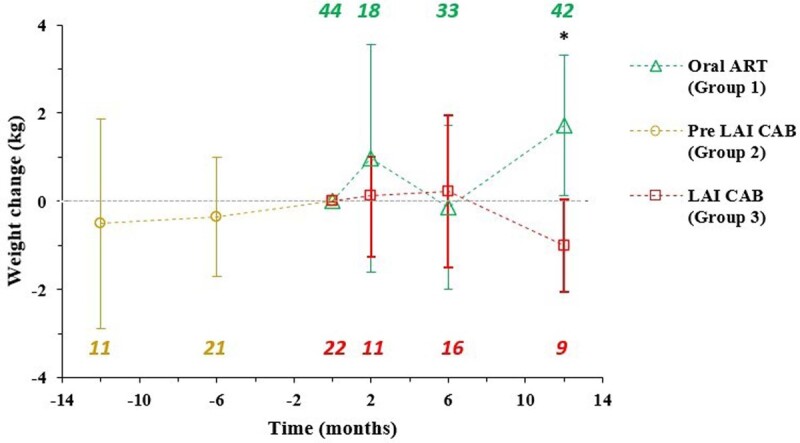

Changes in weight (kg) over time for the three study groups relative to T0, shown here as 0 months. Green triangles represent group 1 (oral INSTI-based regimen), yellow circles represent group 2 (pre-LAI CAB), and red squares represent group 3 (LAI CAB). The asterisk (*) denotes significantly different mean weight changes between groups 1 and 3 (p = 0.0046). Error bars represent 95% confidence intervals around the mean. Italicized numbers are the sample size available for analysis of each group at each given time point.

**Figure:**
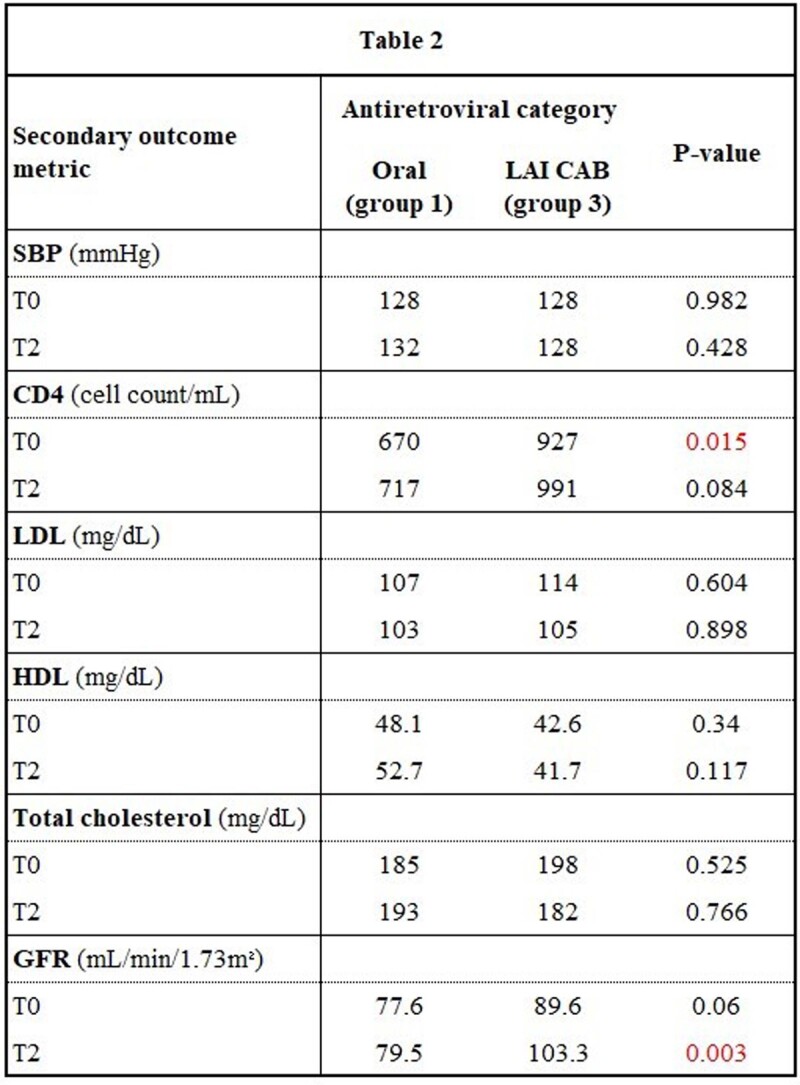

Comparison of secondary outcome metrics between patients taking oral and injectable antiretrovirals. Data were compared between T0 and T2 or between T0 and T1 if no data were available for T2.

**Conclusion:**

Weight loss was observed in a small number of patients who were on LAI CAB relative to patients on oral ART after 12 months. This contrasts with recent clinical trial data showing no significant difference in weight in patients on LAI CAB versus those on oral bictegravir. Our study also found that patients who were on oral ART had lower eGFR after 6 months compared to those on LAI CAB. This may be related to tenofovir exposure, a component of most oral regimens. This represents real-world data outside of a clinical trial, and though limited by size, may impact clinical decision making.

**Disclosures:**

**All Authors**: No reported disclosures

